# Highly porous bio-glass scaffolds fabricated by polyurethane template method with hydrothermal treatment for tissue engineering uses

**DOI:** 10.22038/IJBMS.2022.67272.14746

**Published:** 2022-12

**Authors:** Maryam Badiee, Nahid Hassanzadeh Nemati, Mohammad Taghi Khorasani, Mohammad Ali Shokrgozar

**Affiliations:** 1 Department of Biomedical Engineering, Science and Research Branch, Islamic Azad University, Tehran 14778-93885, Iran; 2 Department of Biomaterials, Iran Polymer and Petrochemical Institute, Tehran 14977-13115, Iran; 3 National Cell Bank of Iran, Pasteur Institute of Iran, Tehran 1316943551, Iran

**Keywords:** Biomedical application, Mechanical strength, Mesoporous structure, Polyurethane foam, Tissue regeneration

## Abstract

**Objective(s):**

Bioglass scaffolds, which contain a significant percentage of porosity for tissue engineering purposes, have low strength. For increasing the strength and efficiency of such structures for use in tissue engineering, fabrication of hierarchical meso/macro-porous bioglass scaffolds, developing their mechanical strength by hydrothermal treatment and adjusting pH method, and achieving the appropriate mesopore size for loading large biomolecules, were considered in this study.

**Materials and Methods:**

Mesoporous bioglass (MBG) powders were synthesized using cetyltrimethylammonium bromide as a surfactant, with different amounts of calcium sources to obtain the appropriate size of the mesoporous scaffolds. Then MBG scaffolds were fabricated by a polyurethane foam templating method, and for increasing scaffold strength hydrothermal treatment (90 ^°^C, for 5 days) and adjustment pH (pH=9) method was used to obtain hierarchical meso/macro-porous structures. The sample characterization was done by Simultaneous thermal analysis, Fourier transform infrared spectroscopy, Field Emission Scanning electron microscopy, small and wide-angle X-ray powder diffractions, transmission electron microscopy, and analysis of nitrogen adsorption-desorption isotherm. The mechanical strength of scaffolds was also determined.

**Results:**

The MBG scaffolds based on 80.28 (wt.) % SiO_2_- 17.89 (wt.) % CaO- 1.81 (wt.) % P_2_O_5_ presented interconnected large pores and pores in the range of 100-150 μm and 6-18 nm, respectively and 0.4 MPa compressive strength.

**Conclusion:**

The total pore volume and specific surface area were obtained from the Brunauer-Emmett-Teller theory, 0.709 cm^3^ g^-1^ and 213.83 m^2^ g^-1^, respectively. These findings could be considered in bone-cartilage tissue engineering.

## Introduction

In the past decades, many kinds of biomaterials were fabricated to promote cell differentiation and enhanced tissue formation to improve tissue engineering. However, finding the material to load growth factors, protection, and the releasing system for the elements for the long life of treatment also remain challenges in tissue engineering. Because of this problem preparing scaffolds with high loading capacity and controlled and sustained release has been noticed by researchers. Recently, biomedical applications of mesoporous bioglasses (MBG) have been critical. MBG showed biocompatibility (without immune reaction after implanting into tissue as biomaterial), bioactivity, degradability, high surface area, ordered mesoporous channel structure for loading factors such as drugs and growth factors, and sustain controlled release behavior. They have been proposed for applications in regeneration medicine and making the treatment more effective and confined to the target area (1-19).

Bioactive glasses were introduced in 1971 by Hench *et al*. (20, 21). Their unique feature is making a chemical bond with hard and soft tissue and avoiding the formation of a fibrous capsule. BGs have commercial applications, including filling osseous cavities, maxilla facial reconstruction, dental applications, coating metallic implants, and tissue engineering (22-27).

Mesoporous Bio-glasses (MBGs) with pore sizes ranging from 2 to 50 nm were introduced in 2004 (28). These materials have the same bioactive glasses (CaO-P_2_O_5_-SiO_2_) composition, but they have superior features, including optimal textural parameters, the ability to load drugs and other molecules, and better bioactivity (29-35). To synthesize the MBGs powders, various surfactants as structure-directing agents have been used, such as cetyltrimethylammonium bromide (CTAB), nonionic triblock copolymer Pluronic (P123), F127, etc. The formation and size of ordered MBGs arrangement was derived from different factors such as concentration and type of surfactant, solvent, pH, temperature, CaO content, calcination temperature, and heating rate (28, 36-43). MBG is a potential biomaterial for the treatment of tissue defects. However, there is a question that remains. For the repair of tissues, the material should be the scaffold to establish new tissue, but the MBG substrate is not the scaffold. Therefore, researchers made MBG scaffolds. The first fabrication of BG scaffolds was pioneered by Chen *et al*. in 2006 (44). The fabrication of macro-porous/mesoporous BG scaffolds is a promising strategy to enhance tissue formation. Macropores provide micro channels for cell migration, nutrition transfer, and tissue in-growth. The mesopores provide the active sites for apatite deposition and loading factors because of their large surface area (9, 10, 13, 14, 45, 46).Although the preparation of macro-porous scaffolds with mesoporous BG has been extensively studied, MBG porous scaffolds with an appropriate mesoporous size for loading growth factors and good mechanical properties need more studies. The development of hierarchical scaffolds based on MBGs may be difficult due to some inherent drawbacks of MBG scaffolds (like high brittleness) and technological challenges related to their fabrication in a multiscale porous form. For example, MBG scaffolds produced by PU foam methods exhibit a low mechanical strength, making them unsuitable for clinical applications. However, these methods are often inexpensive, simple to design, and flexible to optimize physical-chemical properties (7-9, 47). 

In this study, MBGs were synthesized using CTAB surfactant, and the amount of CaO was considered a variable to adjust the size of the mesopores. Cao acts as a modifier of the silica network and influences the mesoporous structure formed after the surfactant removal, then the amount of Cao in MBGs is significant. Some MBGs scaffolds also were fabricated by the polyurethane foam template method (48, 49), and the compressive strengths of the scaffolds were low (<0.045 MPa) (50). The fabricated MBG scaffolds were treated by a hydrothermal method and adjustment pH to obtain macro/mesoporous structures. The same hierarchal structure of course made from TiO_2_ was reported before by Hassanzadeh *et al*. (51). We aimed to increase the mechanical strength of the scaffold and fabricated the scaffold with appropriate mesopores size that can load growth factors. The adsorption capacity of MBGs is controlled by textural properties. Before deciding on loading drugs or other molecules, the diameter of the mesopore and the dimension of a molecule that needs to be loaded should be considered (52, 53). Biomolecules, such as growth factors, have huge dimensions and limit their entrance into mesopores, and only the external surface of the mesoporous bio-glasses is loaded with growth factors. To solve this problem, we use hydrothermal treatment to increase the size of mesopores up to 10 nm. Then these MBGs porous scaffolds can load biological agents for tissue engineering uses. The final aim will be reported next to the writers’ literature. The current work is expected to provide valuable information for the development of MBG scaffolds for tissue engineering, which are potentially able to provide multiple therapeutic actions by supporting tissue ingrowth and regeneration while simultaneously providing local treatment of diseased tissue or accelerating tissue formation through the controlled release of organic drugs and growth factors. The manufactured samples and MBGs powders were characterized using Field Emission scanning electron microscopy (FE-SEM), X-ray diffraction (XRD), Fourier transform infrared (FTIR), nitrogen adsorption/desorption isotherms, and transmission electron microscopy (TEM). Testing the compressive strength of the samples determined the mechanical strengths of the scaffolds.

## Materials and Methods

***Experimental Procedure***

***Materials***

Materials used in this work including tetraethyl orthosilicate (TEOS (98%), C_8_H_20_O_4_Si, No. 800658), triethyl phosphate (TEP (99%), C_6_H_15_O_4_P, No. 8.21141), hexadecyltrimethylammonium bromide (CTAB, C_19_H_42_BrN, No. 814119), calcium nitrate tetrahydrate (Ca(NO_3_)_2_.4H_2_O (99%), No.1.02121), and ethanol (EtOH (99.7%), C_2_H_5_OH, No. 100983) were purchased from the Merck co. Ammonium hydroxide solution (32 wt% in H_2_O, NH_4_OH, No. 318612) was purchased from Sigma Aldrich Co. For prepared scaffolds, PU foam (PUF) (poret^®^ polyester, cell counts PPI 60, 80 Dunckel) was supplied by EMW. The foam is a kind of sponge that has open and interconnected porosity and liquid-absorbing properties.

***Synthesis of mesoporous bio-glass powders***

The mesoporous bioglass powder synthesis using the evaporation-induced self-assembly (ELISA) method was carried out at different amounts of Ca(NO_3_)_2_. 4H_2_O as listed in Table 1 (37-39, 53). The structure-directing agent in this synthesis was CTAB (cationic surfactant). In a typical synthesis of MBG (1), 165 ml deionized water was mixed with 78 ml absolute ethanol at 25 ^°^C under magnetic stirring (400 rpm) for 15 min. Then 0.345 g CTAB and 2 ml ammonium hydroxide solution were added with a time interval of 30 min along with non-stop stirring. The surfactant was added to create mesostructure and spherical powder structure. After that 3 ml, of TEOS, was added to Sol and stirred for 90 min. By adding TEOS, the color of the Sol began to be opaque due to hydrolysis reactions. In the end, 0.23 ml TEP and 0.64 g Ca(NO_3_)_2_. 4H_2_O were added with a time interval of 30 min, and the whole Sol was stirred for five hours. Next, the Sol was centrifuged and washed with deionized water and ethanol (the ratio was 2:1) for 20 min, 10000 rpm, and repeated this step 3 times (Laboratory centrifuge, Sigma 4K15C, Germany). After centrifugation, samples were placed in an oven at 80 ^°^C for 24 hr (Memmert, GmbH+Co.KG, Germany). The obtained powders were washed with deionized water and filtrated and dried at room temperature. In the end, powders were calcined at 600 ^°^C for 5 hr (2 ^°^C/min) in a furnace (EX, 1300-33l, Exciton, Iran) for removing the surfactant and form the final MBG powders (Si/Ca/P molar ratio:85/14.88/0.1). For comparison, sample without CTAB (BG) was also synthesized above the mentioned process.

***Scaffold preparation***

**Figure 1:**
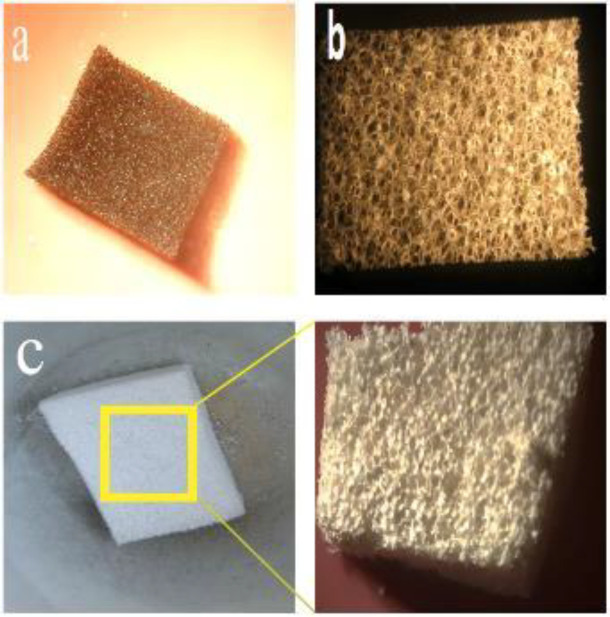
Photographs of the template of (a) PUF, (b) PUF after dipping in MBG solution, and (c) MBG scaffolds after calcination at 600 ^°^C for 5 hr (2 ^°^C / min)

PUF: Polyurethane foam; MBG: Mesoporous bio-glass

In all three samples of synthesized mesoporous bioglass powders, increasing pore volume and specific surface area were observed, compared with the BG sample without CTAB surfactant (Table 2). The purpose of this study was to build a scaffold with the ability to load growth factors. MBG (2) (Ca/P/Si=18/2/80 molar ratio) due to its suitable pore size (9.46 nm) and specific surface area (213.83 m^2^/g) was selected. For the preparation scaffold with greater strength and solution stability, hydrothermal treatment was used. To prepare the Sol, all steps mentioned in synthesis MBG (1) were repeated except the final stage (adding Ca(NO_3_)_2_.4H_2_O), the Sol was heated at 90 ^°^C (heating rate 1 ^°^C/min) in a water bath. Then Ca(NO_3_)_2_.4H_2_O was added, pH (pH=9) adjusted, and continuously stirred for 5 days. We were afterward turning off the stir and waiting for cooling the Sol to room temperature. PU foams with a dimension of 10 mm × 10 mm × 10 mm (immersed, washed with deionized boiling water (to release mechanical stress stored in the foam), and let dry) were completely immersed into the Sol and compressed to force the Sol to migrate into the pores (the time of immersion was 10 min). After being immersed, the sponges were moved to a petri dish and allowed to volatilize for one day at room temperature, and this step was repeated eight times for samples. After the porous scaffolds were dried at room temperature, they dried at 80 ^°^C for 24 hr in the oven. In the end, for the removal of PU foam and surfactant and to increase the strength of the scaffolds, they were calcined in a vacuum furnace (heating rate 2 ^°^C/min, 5 hr). The scaffolds were cooled in the furnace by turning off the furnace. After heating and cooling, the MBG scaffolds were washed with acetone and air dried. Figure 1 shows the optical photographs of the template of PUF, PUF after dipping in MBG solution, and MBG scaffolds after calcination.

***Characterizations***

The chemical composition of the prepared MBG was obtained from X-ray Fluorescence Spectroscopy (XRF PHILIPS PW1410, the Nether Lands) analysis. The thermal decomposition temperature of PUF and surfactant was determined by a simultaneous thermal analysis (STA) test, which was performed from room temperature up to 800 ^°^C (heating rate 10 ^°^C/min, N2 atmospheric environment). For comparison, STA analysis was performed in the presence and absence of PUF and surfactant. Table 2 shows the designation of each sample. The morphologies, structures, pore connectivity, pore size, and elemental composition of the MBG powders and scaffolds were observed by field emission scanning electron microscopy coupled with an Oxford energy dispersive spectroscopy analysis system (FE-SEM TESCAN MIRA3, Czech Republic). Before imaging, the powders and scaffolds were sputtered with a thin layer of gold (NSC, model: DSR1). To measure the size and morphology of MBG powders and observe mesostructure transmission electron microscope (TEM ZEISS EM 900, Germany) and Scanning electron microscope (FEI Quanta 200 SEM) were used. The chemical composition and crystalline and amorphous structure of MBGs were investigated by wide-angle X-ray diffraction (WAXRD, X’ pert PRO MPD Panalytical, the Netherlands). To study order structured in MBG, Low-angle XRD (2θ=0.5-8^°^) was used at a scanning speed of 1 ^°^/min. The pore volume, specific surface area, and distribution of mesopores were calculated by the Brunauer-Emmett-Teller method (BET) and Barret-Joyner-Halenda (BJH) method, respectively. Detecting the amount of nitrogen gas adsorbed and desorbed by the surface of samples was done at a constant temperature of liquid nitrogen (77 K). To prepare and dry the samples, they were degassed under the temperature of 120 ^°^C for 15 hr. Fourier transform infrared spectroscopy (FTIR, Thermo Nicolet Nexus 870) was used to determine the chemical composition and identification of functional groups. To prepare samples, the powder of samples was mixed with reference material (KBr) and made into a pill, and placed under light irradiation. For each sample, transmission spectra were measured in the range of 400-4000 cm^-1^.

***Mechanical test***

Compressive strength tests were done to determine the compressive strength of the MBG scaffolds. Scaffolds with a dimension of approximately (5 mm × 7 mm × 0. 5 mm) were selected. They were placed between two parallel plates and compressed (Cross-head speed: 0.001 mms^-1^). The peak of the stress-strain curve indicated compressive strength

## Results

***XRF analysis***

The chemical composition of the prepared MBG powders, calcined at 600 ^°^C for five hr (2 ^°^C/min), obtained from XRF analysis is shown in Table 3. The MBG powders are composed of SiO_2_, CaO, and P_2_O_5_.

***Thermal Analysis***

**Figure 2:**
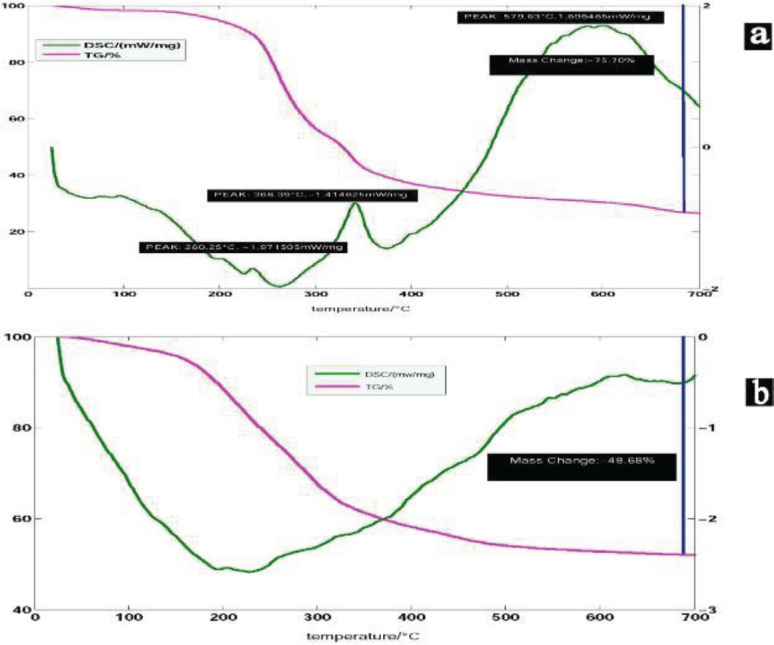
STA analysis of (a) SFMBG (75.70% weight loss, two exothermic peaks at 368.39 ^°^C and 579.63 ^°^C are related to the exit of foam and surfactant, respectively) and (b) SNMBG (48.68 % weight loss due to elimination of water, ethanol, and decomposition of Ca(NO_3_)_2_.4H_2_O) samples

STA: Simultaneous thermal analysis; SFMBG: Sample containing surfactant CTAB and Polyurethane foam; SNMBG: Sample without surfactant CTAB and Polyurethane foam

Simultaneous Thermal Analysis (STA) was performed to study the weight loss and determine the temperature of surfactant and PUF removal of the calcination step of MBG scaffold synthesis. Figure 2a is related to the sample containing surfactant CTAB and PUF (SFMBG). It showed 75.70% weight loss-two exothermic peaks at 368.39 ^°^C and 579.63 ^°^C related to the exit of foam and surfactant, respectively. After 600 ^°^C, no significant weight loss was observed, which indicates that the calcination process was completed at 600 ^°^C. The calcination temperature was not selected higher than 600 ^°^C for maintaining the mesoporous structure. Figure 2b, which is related to the sample without surfactant CTAB and PUF (SNMBG), showed a 48.68% weight loss due to elimination of water, ethanol, and decomposition of Ca(NO_3_)_2_.4H_2_O. 

***FE-SEM Analysis***

**Figure 3:**
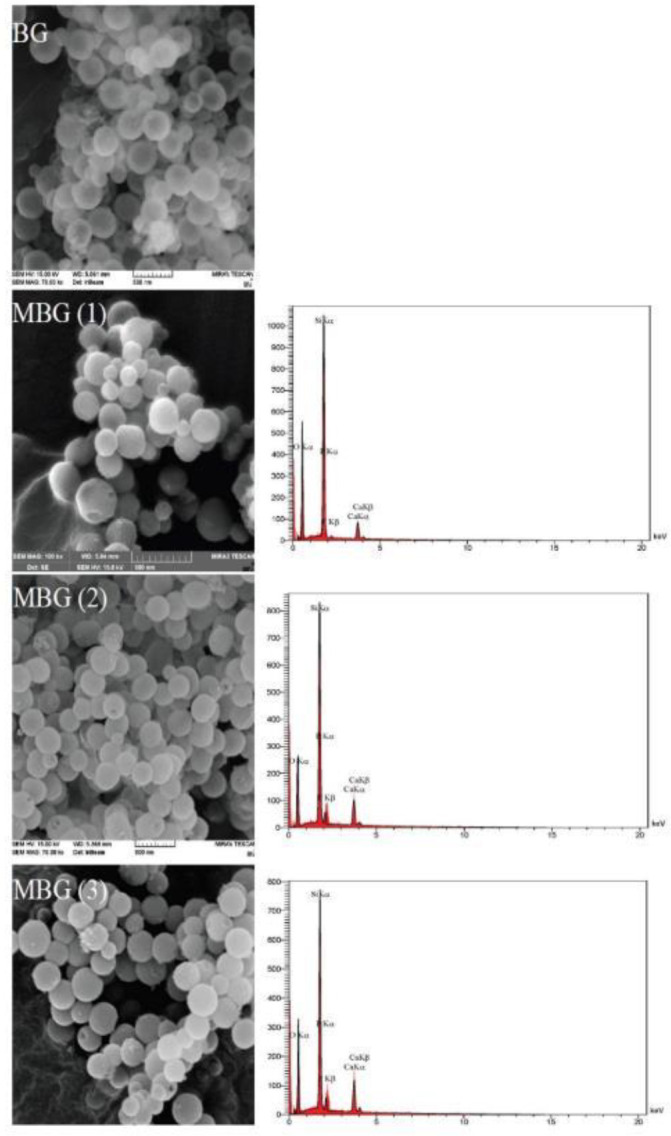
FE-SEM micrographs of synthesized MBG powders with different amounts of Ca(NO_3_)_2_.4H_2_O and BG without surfactant and EDS spectra of MBG powders

**Figure 4:**
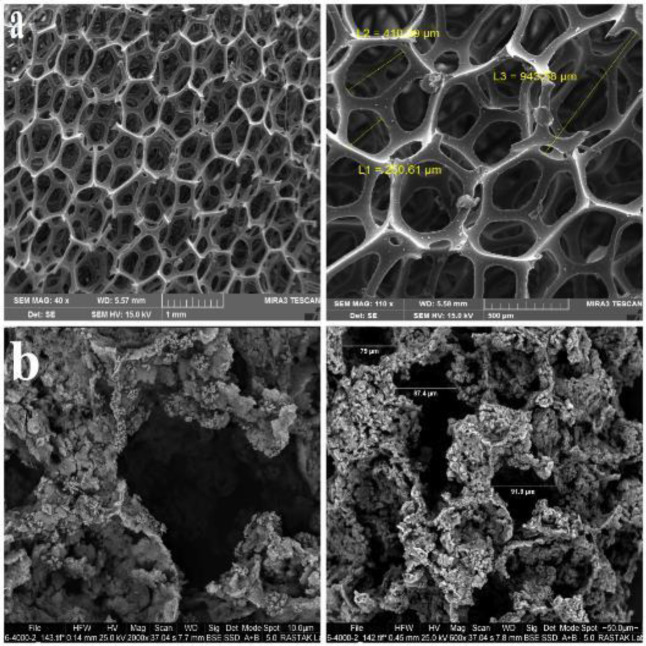
SEM micrographs of (a) PUF at different magnifications 40 x, 110 x and (b) MBG scaffolds at different magnifications 600 x, 2000 x

PUF: Polyurethane foam; MBG: Mesoporous bio-glass

**Figure 5:**
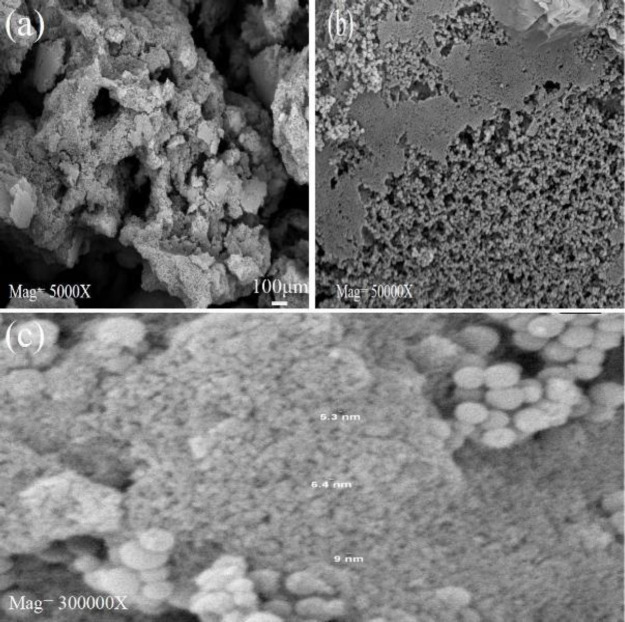
SEM micrographs of mesopores in the fabricated MBG scaffolds at different magnifications (a) 5000 x, (b) 50,000 x, and (c) 300,000 x

The morphology, size, and mesostructure of synthesized MBG powders with different amounts of Ca(NO3)_2_.4H_2_O and BG without surfactant are shown in Figure 3. All MBG samples exhibited regularly spherical and homogeneous morphology with a hole in their surface. This hole indicated the mesoporous, which had a size range between 37-47 nm and not a hole exhibited on the surface of the spherical BG sample. All MBG powders showed narrow particle size distribution and good dispersibility. The elemental composition of synthesized MBG powders is characterized by energy dispersive spectroscopy (EDS) analysis. Figure 3 shows the elemental composition of MBG (1), MBG (2), and MBG (3). Silicon (Si), calcium (Ca), phosphorous (P), and oxygen (O) were detected in all samples. Changes in peak intensity were due to differences in the amount of calcium sources. Figure 4a Shows the morphology of macro pores of PUF that are interconnected. Figure 4b shows the morphology of macro pores of the MBG scaffold after calcination at 600 ^°^C for 5 hr with a heating rate of 2 ^°^C/min. The mean diameter of pores was in the range of 50-150 μm. Compared with the initial pore diameter of PUF around (500 µm), the macro pore diameter of the scaffolds decreased to around (150 µm). This decrease is related to the shrinkage incurred during the calcination process. Figure 5a, b shows the formation of mesoporous structure in the walls of scaffolds after calcination at 600 ^°^C for 5 hr. In fact, the mesopores are formed in the arm of the macropores of the scaffold. The size of mesopores is the same as the results obtained from BET analysis and according to the definition of mesopores (up to 50 nm) (Figure 5c).

***TEM Analysis***

**Figure 6:**
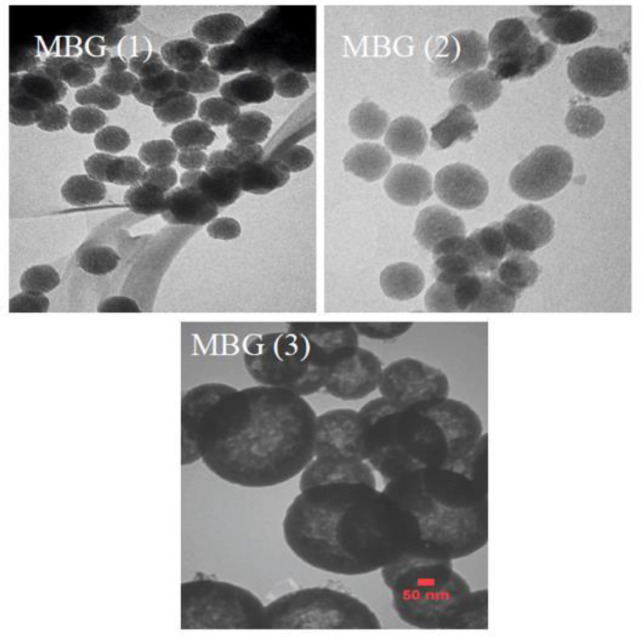
TEM image for MBG powders prepared under different Ca(NO_3_)_2_.4H_2_O concentrations with well-ordered mesostructure

TEM analysis for synthesized mesoporous bio-glasses prepared under different Ca (NO_3_)_2_.4H_2_O concentrations showed a well-ordered hexagonal and uniform mesoporous structure. Mesoporous size could be tuned by changing the amount of Ca(NO_3_)_2_.4H_2_O. The spheres become solid in character when the amount of Ca(NO_3_)_2_.4H_2_O was further increased to 2.1 g (MBG (3)). The MBG particles exhibited spherical geometry and uniform size and there were a great number of mesopores inside the MBG nanospheres (Figure 6). 

***BET-BJH Analysis***

**Figure 7:**
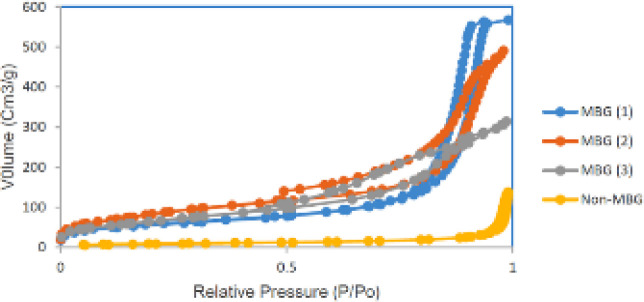
Nitrogen adsorption-desorption isotherm of MBG powders with different contents of Ca(NO_3_)_2_ .4H_2_O and non-MBG powders

**Figure 8:**
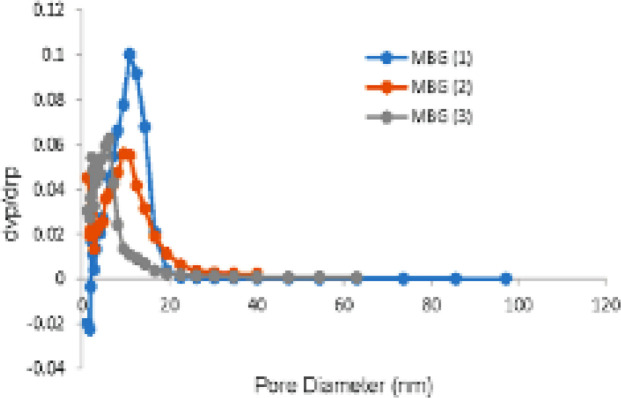
Mesoporous size distribution of synthesized MBG powders

The nitrogen sorption isotherms and pore size distribution curves are shown in Figure 7, and textural parameter values obtained from this test are listed in Table 4. All synthesized MBG samples indicated type IV isotherm patterns (exhibited characteristics of the type IV isotherm of mesoporous materials according to IUPAC classification) which are a mesoporous property with H3 hysteresis loop, which indicates that mesoporosity is slot shaped (54). However, in the samples without surfactant (CTAB), no mesoporous structure was observed. Therefore, it did not exhibit typical isotherms. With a change in the amount of Ca sources, specific surface area and pore volume changed. With an increasing amount of Ca(NO_3_)_2_. 4H_2_O specific surface area increased and pore volume decreased. The pore size distribution curve is derived from the adsorption branch of isotherms, which represents the narrow range of porosity and monomodal distribution. A decrease in mesopore size was observed with increasing Ca because of silicate network de-polymerization. Figure 8 shows the narrow size distribution (about 6-18 nm) of mesopores. The mesopore size distribution can be tuned by controlling the processing parameters (PH and temperature) and Sol compositions. The size of the mesoporous is an essential parameter for loading biomolecules and drugs. Loading small molecules and drugs in mesoporous bio-glasses would not have any problem. However loading more giant molecules such as peptides, growth factors, and some drugs like metal-based drugs would have the problem of penetrating these molecules into the mesoporous structure. The external surface of the mesoporous bio-glasses only adsorbed these molecules (52, 53). In this study, to solve this problem, we changed the amount of Ca(NO_3_)_2_. 4H_2_O and used hydrothermal treatment to achieve a large mesoporous structure (6-18 nm) suitable for large molecules. The mesoporous texture, high surface area (213.83 m^2^g^-1^), and high pore volume (0.709 cm^3^g^-1^) of synthesized bio-glasses allow hosting of many bio-molecules in their structure. Tailoring the mesoporous size would modulate the release kinetics of biomolecules.

***XRD Analysis***

**Figure 9:**
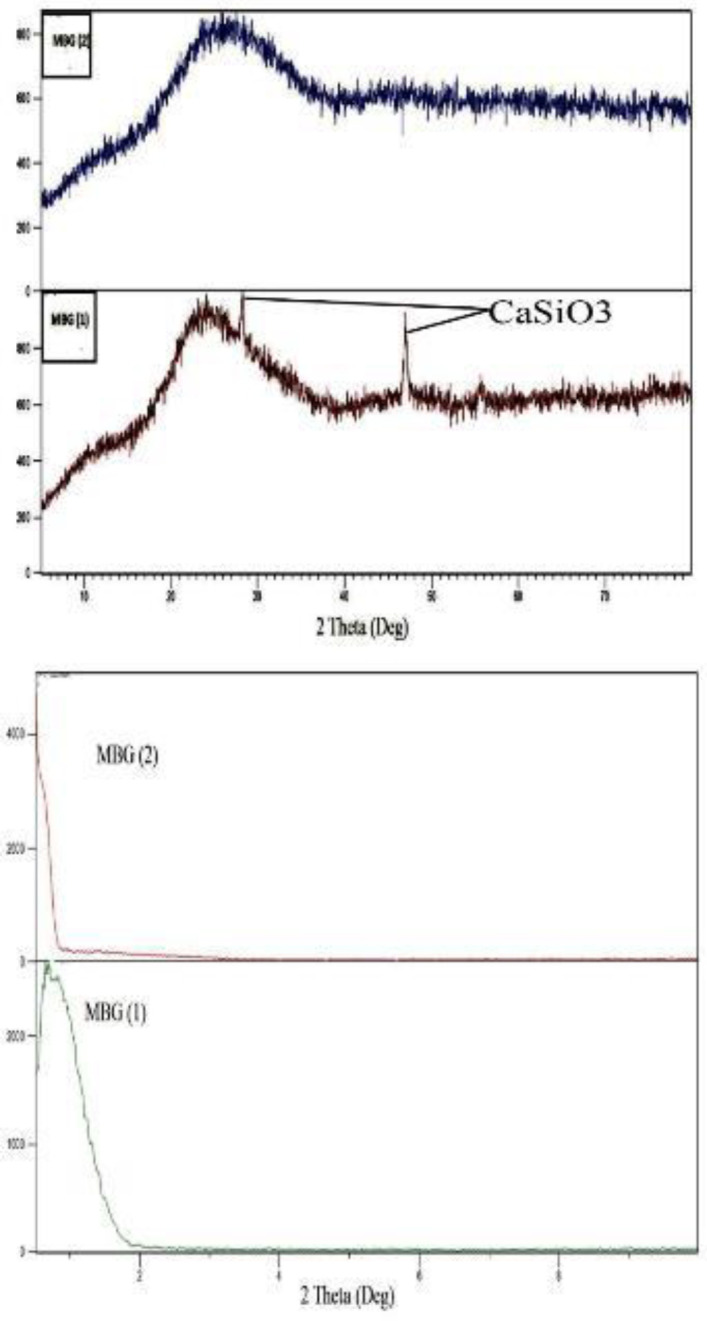
(a) Wide-angle XRD analysis for synthesized MBG powders with different contents of Ca(NO_3_)_2_.4H_2_O after calcination at 600 ^°^C for 5 hr and (b) Low-angle XRD analysis for synthesized MBG powders

XRD: X-ray diffraction; MBG: Mesoporous bio-glass

Figure 9 (a) shows the patterns of synthesized MBG powders with different content of Ca (NO_3_)_2_.4H_2_O after calcination at 600 ^°^C for five hours. In MBG (1), Bragg’s reflection was seen, corresponding to the crystalline formation of CaSiO_3_. In other samples, no distinct diffraction peaks appeared. The broad diffraction at about 20-37 ^°^(2θ) is associated with mesoporous bio-glasses characteristic amorphous nature and indicates that the synthesized MBG powders were amorphous. Low-angle XRD patterns for the 2θ angle ranging from 0^°^ to 8^°^ for synthesized MBG powders are shown in Figure 9 (b). There are apparent diffraction peaks around 2θ=0.8^°^ appeared. The peaks at low angles indicate the ordered mesoporous channels presented in samples.

***FTIR Analysis***

**Figure 10:**
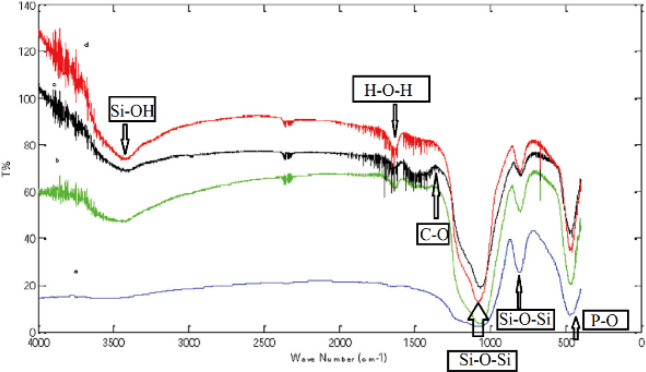
FT-IR spectra of the MBG powders with different amounts of Ca(NO_3_)_2_.4H_2_O (a) BG, (b) MBG (1), (c) MBG (2), and (d) MBG (3)

BG: Bio-glass; MBG: Mesoporous bio-glass

The FTIR spectra of the synthesized MBG powders with different amounts of Ca sources (Figure 10) showed an adsorption band at 1069 cm^-1^, 798 cm^-1^, and 467 cm^-1^. This adsorption is related to the stretch vibration and bending vibration of Si-O-Si. The peaks at 3438 cm^-1^ were related to the Si-OH group. As the amount of calcium source increased, the intensity of the peak at 1090 cm^-1^ increased. 

***Mechanical Analysis***

**Figure 11:**
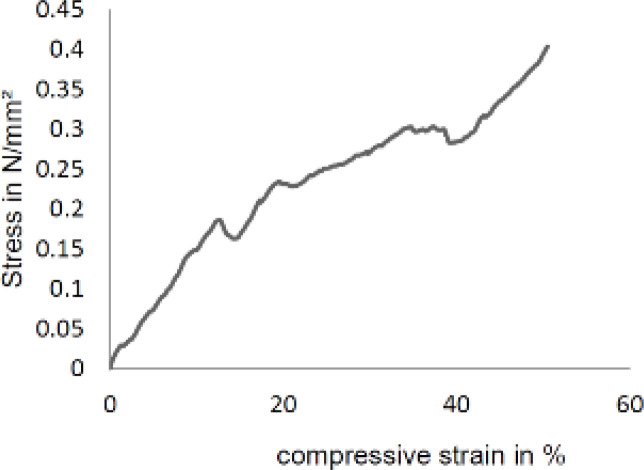
Compressive stress-strain curves of MBGs scaffold fabricated by the polyurethane foam template and treated by a hydrothermal method

Mesoporous bioglass ceramic scaffolds without hydrothermal treatment and the use of a vacuum furnace, as in the references and as was expected, had a low compressive strength. They could not be moved easily by hand (compressive strength: <0.045 MPa) (50). After heat treatment and adjustment of pH to 9, the compressive strength of MBG scaffolds increased (compressive strength: 0.4 MPa) (Figure 11). By this method, it could be able to preserve the mesoporous structure of scaffolds and achieve greater strength at low temperatures than other ceramic MBG scaffolds. The compressive strength of the fabricated scaffold was almost like the compressive strength of the polymer-coated (PDLLA) bio-glasses scaffold made by the polyurethane template method (50). This increase in compressive strength is the increase in the stability of the mesoporous bio-glass solution, which was achieved by applying heat treatment and pH adjustment. In fact, after immersion of polyurethane foam as a template, mesoporous bio-glass solution uniformly covers the outer and inner surfaces of the foam. Uniform coating of the solution on the inner and outer surfaces of the foam causes the inside of the bioglass scaffold obtained not to have large cavities, after removing the polyurethane foam during the calcination process. Mesoporous bioglasse particles are close to each other to create a bond after removing the polyurethane foam.

**Table 1:** Amounts of reactants used for synthesis of MBG powders in different amounts of Ca(NO_3_)_2_.4H_2_O

**Samples**	**Anhydrous ethanol (C**_2_ **H**_6_ **O)**	**Ammonium hydroxide solution** ** (NH**_4_ **OH, 5.0 M)**	**Triethyl phosphate (TEP, 99%)** **C**_6_ **H**_15_ **O**_4_ **P**	**Tetraethyl orthosilicate** ** (TEOS, 99%)** **C**_8_ **H**_20_ **O**_4_ **Si**		**Calcium nitrate-tetra hydrate (Ca(NO**_3_ **)**_2 _ **.4H**_2_ **O)**	**Surfactant (CTAB)**	
MBG (1)	78 ml	2.34 ml	0.23 ml	3 ml	0.64 g		0.345 g
MBG (2)	78 ml	2.34 ml	0.23 ml	2.4 ml	1.57 g		0,345 g
MBG (3)	78 ml	2.34 ml	0.23 ml	2.1 ml	2.1 g		0.345 g
BG	78 ml	2.34 ml	0.23 ml	3 ml	0.64 g		-

MBG: Mesoporous bioglass

**Table 2:** Designation of samples in the presenc e and absence of PU foam and surfactant

**Designation**	** PU Foam **	**(NH**_4_ **OH, 5.0 M)**	**C**_6_ **H**_15_ **O**_4_ **P**	**C**_8_ **H**_20_ **O**_4_ **Si**	** (Ca(NO**_3_ **)**_2_ **4H**_2_ **O)**	**Surfactant (CTAB)**
SNMBG	×	2.34 ml	0.23 ml	3 ml	0.64 g	×
SFMBG	√	2.34 ml	0.23 ml	3 ml	0.64 g	√

√: in the presence of specific material; ×: in the absence of specific material

PU: Polyurethane

**Table 3:** Composition of the MBG powder obtained from XRF analysis

Samples	SiO_2_ (%)	CaO (%)	P_2_O_5_ (%)
MBG (1)	85.031	14.886	0.104
MBG (2)	80.288	17.897	1.815
MBG (3)	76.482	21.343	2.175

XRF: X-ray Fluorescence; MBG: Mesoporous bio-glass

MBG: Mesoporous bio-glass

MBG: Mesoporous bio-glass

**Table 4:** Effect of Ca sources content on the specific surface area, pore volume, and pore size of MBG powders after calcination at 600 ^°^C for 5 hr

**Samples **	**S**_BET_ ** (m** ^2^ **/g)**	**V**_P_ ** (cm** ^3^ **/g)**	**Pore size (nm)**
MBG (1)	158.05	0.859	10.90
MBG (2)	213.83	0.709	9.46
MBG (3)	201.28	0.463	6.23
Non-MBG	28.765	0.205	1.21

S_BET_: BET surface area of powders; V_P_: Pore volume of powders

MBG: Mesoporous bio-glass

MBGs: Mesoporous bio-glasses

## Discussion

The mesoporous structure of MBG scaffolds is important for biomolecule loading and delivery. They are many developed methods to obtain mesoporous bio-glasses but the size of the mesoporous structure is a very important parameter for loading biomolecules and drugs. Loading small molecules and drugs in mesoporous bio-glasses would not have any problem but loading bigger size molecules such as peptides, growth factors, and some drugs like metal-based drugs would have a problem penetrating these molecules into the mesoporous. The external surface of the mesoporous bio-glasses only adsorbed these molecules (53). In this study to solve this problem, we changed the amount of Ca(NO_3_)_2_4H_2_O and used hydrothermal treatment to achieve a large mesoporous size (6-18 nm) that is suitable for large molecules. By polyurethane template method with hydrothermal treatment, MBG scaffolds with hierarchically large pores (50-150 μm) and well-ordered mesopores (6-18 nm) were obtained. The use of the heat treatment method in the synthesis of mesoporous bio-glasses increased the compressive strength of the scaffold (0.4 MPa) in comparison with similar scaffolds made by the polyurethane template method (<0.045 MPa) (50). The compressive strength of the fabricated scaffold was almost similar to the compressive strength of the polymer-coated (PDLLA) bio-glasse scaffold made by the polyurethane template method (50).

The manufactured MBG scaffolds still maintained a well-ordered mesoporous structure, as well as a high surface area (213.83 m^2 ^g^-1^) and high pore volume (0.709 cm^3 ^g^-1^), all of which are important for the loading and delivery of a greeting number of bio-molecules. Actually, tailoring the mesoporous size would modulate the release kinetics of biomolecules.

## Conclusion

Meso/Macroporous bio-glass scaffolds were synthesized by the polyurethane foam template method with hydrothermal treatment and adjustment of pH=9. To achieve a scaffold with the ability to load the growth factor, Ca(NO_3_)_2_.4H_2_O=1.57 g was selected. CTAB surfactant and polyurethane foam templates (60, 80 PPI) were used to fabricate the MBG scaffold. The result showed that the MBG scaffold presented interconnected macro pores and mesoporous walls due to elimination of PUF, in the range of (100-150 µm) and elimination of surfactant, in the range of (6-18 nm), respectively. The mechanical properties (compressive strength) of the scaffold were improved by this fabrication method (0.4 MPa). Therefore, these results demonstrate that the MBG scaffolds provide a potential application as a carrier of growth factor in tissue engineering by considering the mechanical properties, controllable architecture, and mesoporous and macroporous and cell attachment capabilities of these scaffolds.

## Authors’ Contributions

BM helped with funding acquisition, visualization, writing original draft preparation, resources, and investigation; HNN provided project administration, supervision, validation, analysis and interpretation of data, and decision to submit the paper for publication; KMT provided methodology and conceptualization; SMA provided supervision and methodology.

## Funding

This research did not receive any specific grant from funding agencies in the public, commercial, or not-for-profit sectors.

## Conflicts of Interest

All authors declare that they have no conflicts of interest.
